# Identification of pathogenic genes and transcription factors in respiratory syncytial virus

**DOI:** 10.1186/s12887-020-02480-4

**Published:** 2021-01-08

**Authors:** Lei Li, Yong An Ni, Zhenfeng Song, Zhi Yi, Fang Wang

**Affiliations:** Department of Pediatrics, The Affiliated Hospital of Qingdao University, No.1677, Wutaishan Road, Huangdao Distict, Qingdao, 266555 China

**Keywords:** Respiratory syncytial virus, Transcription factor, Differentially expressed gene, Integrated analysis, GEO, Biomarker

## Abstract

**Background:**

Respiratory syncytial virus (RSV) is a major cause of acute lower respiratory infections in children, especially bronchiolitis. Our study aimed to identify the key genes and upstream transcription factors in RSV.

**Methods:**

To screen for RSV pathogenic genes, an integrated analysis was performed using the RSV microarray dataset in GEO. Functional annotation and potential pathways for differentially expressed genes (DEGs) were further explored by GO and KEGG enrichment analysis. We constructed the RSV-specific transcriptional regulatory network to identify key transcription factors for DEGs in RSV.

**Results:**

From three GEO datasets, we identified 1059 DEGs (493 up-regulated and 566 down-regulated genes, FDR < 0.05 and |Combined.ES| > 0.8) between RSV patients and normal controls. GO and KEGG analysis revealed that ‘response to virus’ (FDR = 7.13E-15), ‘mitochondrion’ (FDR = 1.39E-14) and ‘Asthma’ (FDR = 1.28E-06) were significantly enriched pathways for DEGs. The expression of IFI27, IFI44, IFITM3, FCER1A, and ISG15 were shown to be involved in the pathogenesis of RSV.

**Conclusions:**

We concluded that IFI27, IFI44, IFITM3, FCER1A, and ISG15 may play a role in RSV. Our finding may contribute to the development of new potential biomarkers, reveal the underlying pathogenesis and also identify novel therapeutic targets for RSV.

## Background

Globally, respiratory syncytial virus (RSV) is the major pathogen of acute lower respiratory infections in children under 5 years old [1–3]. RSV also is an severe virus in adults, causing 25% of lethal respiratory infections in cold seasons, similar to the rate for seasonal influenza [4]. Morbidity caused by RSV may also be long-term, children hospitalized with RSV are more likely to suffer from asthma, sometimes for years, after acute infection [5].

Similar to other respiratory viruses, RSV infection is absorbed by airway epithelial cells, alveolar macrophages, and intraepithelial dendritic cells, induces direct antiviral responses through cytokines and chemokines, and initiates adaptive immune responses [6]. The severity of RSV infection is partly due to currently known risk factors, including medical complications and young age [7]. However, most infants hospitalized for respiratory syncytial virus infection have reportedly been previously healthy and have no risk factors for serious illness [8, 9]. Therefore, currently known risk factors do not fully explain the significant variability of the severity of the disease. Thus, it is important to find biomarkers related to the diagnosis of RSV.

In our study, we performed an integrated analysis of three gene expression datasets to identify the DEGs and transcription factors (TFs) associated with RSV. We identified the differentially expressed genes (DEGs) and TFs of RSV in this integrated analysis. Functional annotation and PPI network construction were performed to explore the biological function of DEGs. Our purpose is to provide clues to reveal the underlying mechanism of RSV and further develop potential new diagnosis and treatment for RSV.

## Methods

### Microarray expression profiling in GEO and identification of DEGs in RSV

The gene expression profiles of children RSV were gained from GEO database with following key search terms: (“respiratory syncytial viruses”[MeSH Terms] OR Respiratory syncytial virus [All Fields]) AND “Homo sapiens”[porgn] AND “gse”[Filter]. Datasets meet the following criteria would be included in our study: (1) selected datasets should be whole-genome mRNA expression profile by array; (2) these data were derived from blood samples of patients with RSV and normal controls; (3) datasets were normalized or original.

After downloading the selected datasets, we deleted the undetectable gene (ie, the genes whose expression value was less than 0 was more than 20% of the total sample size). There were 8834 genes in the intersection of the three datasets. For each dataset, log2 is converted to scale standardization. MetaMA was applied to obtain the DEGs. Genes with FDR < 0.05 and |Combined.ES| > 0.8 were selected as DEGs.

### Functional annotation of DEGs and PPI network construction

GeneCoDis3 was employed to perform GO and KEGG pathway enrichment analysis. The threshold of FDR < 0.05 was considered as significant. Top 50 up- and down-regulated DEGs were searched with the BioGrid, and PPI network was constructed with Cytoscape software.

### Construction of TF regulatory network

With UCSC Genome Bioinformatics (http://genome.ucsc.edu), the corresponding promoters of the top 20 up-regulated or down-regulated DEGs were acquired. Transcription factors (TFs) involved in regulating these DEGs were collected from the match tools in TRANSFAC. The transcriptional regulatory network was visualized by using Cytoscape software.

### QRT-PCR confirmation

We collected blood samples from three RSV patients and three healthy children, and RNA samples were isolated from which to verify the expression level of candidate genes using qRT-PCR. The clinical characteristics of individuals included in this study were displayed in Table S1. We obtained the written informed consent from every participant and the approval from the ethics committee of The Affiliated Hospital of Qingdao University (QYFYW2LL25724). The human 18srRNA was used as endogenous control in analysis.

### Validation in the GEO dataset and receiver operating characteristic (ROC) analysis

GSE34205, GSE38900, GSE42026 and GSE105450 were downloaded from GEO database. GSE34205 performed on GPL570, including 22 healthy controls and 51 RSV. GSE38900 performed on GPL10558, including 8 healthy controls and 28 RSV. GSE42026 performed on GPL6947, including 33 healthy controls and 22 RSV. GSE105450 performed on GPL10558, including 38 healthy controls and 89 RSV. The same data processing was performed for these four datasets as for the integration analysis. The expression levels of selected DEGs were validated with these four datasets. Then, by using pROC package in R language, we performed the ROC analysis to assess the diagnostic value of DEGs. The area under the curve (AUC) was further calculated.

## Results

### Differential expression analysis of genes in RSV

**Fig. 1:**
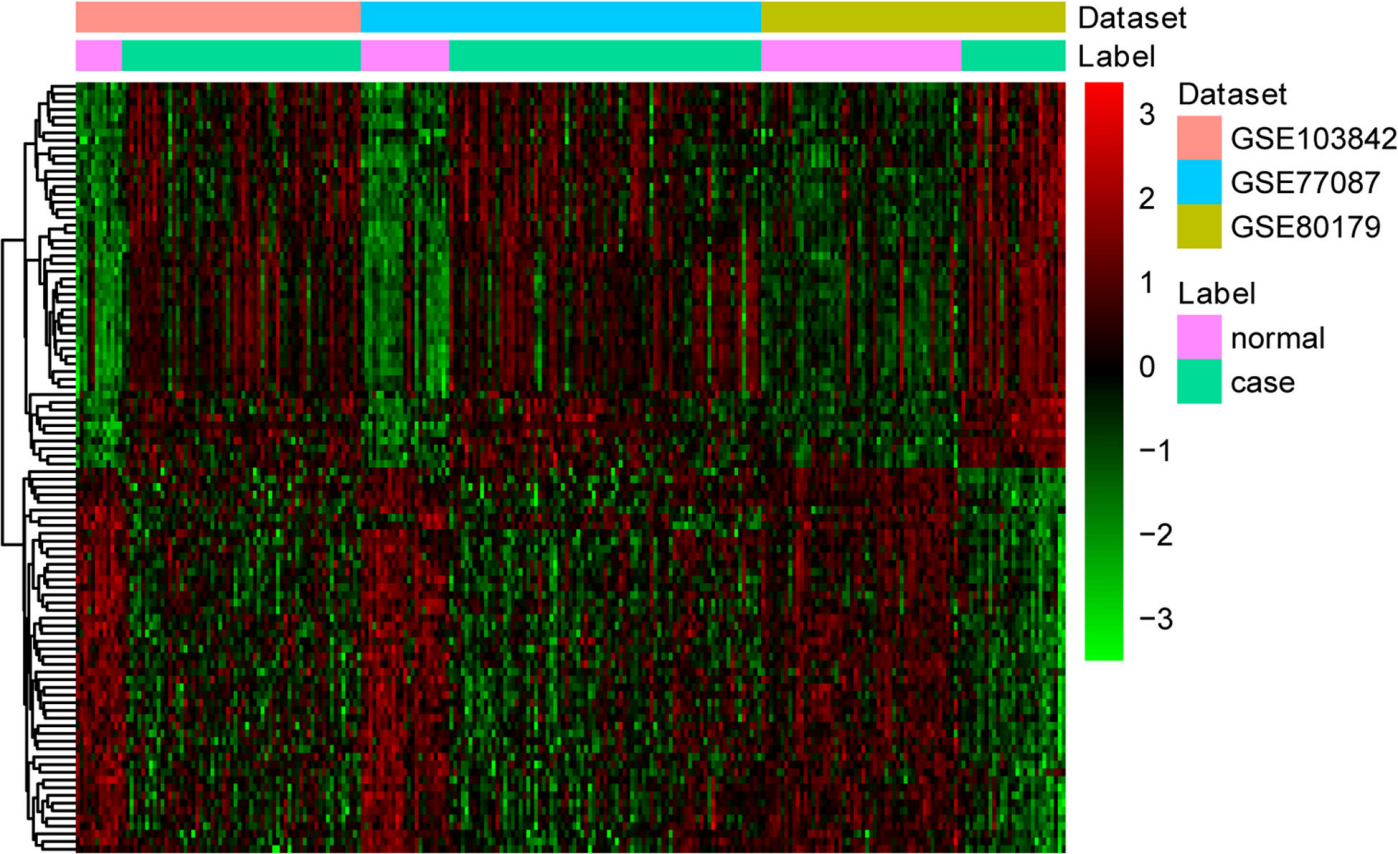
Heatmap image displaying genes that were significantly up-regulated or down-regulated (FDR < 0.05, |Combined.ES| > 0.8) in RSV compared to normal controls

After filtering, a total of three datasets (GSE103842, GSE80179 and GSE77087) were retained for the analysis, the details of these three datasets were shown in the Table 1. A principal component analysis (PCA) of these three datasets was performed (Figure S1). By integrated analysis, 1059 DEGs (493 up- and 566 down-regulated) were obtained in RSV with FDR < 0.05 and |Combined.ES| > 0.8. Among them, IFI27 and MEGF6 was the most up- and down-regulated genes, respectively (Table 2). The heatmap of top 100 up- and down-regulated DEGs produced by cluster analysis is shown in Fig. 1.

**Table 1:** The details of included three datasets

GEO accession	Author	Platform	Samples (P: N)	Year	Tissue
GSE103842	Nicole Baldwin	GPL10558 Illumina HumanHT-12 V4.0 expression beadchip	62:12	2017	blood
GSE80179	Myrsini Kaforou	GPL10558 Illumina HumanHT-12 V4.0 expression beadchip	27:52	2016	blood
GSE77087	Nicole Baldwin	GPL10558 Illumina HumanHT-12 V4.0 expression beadchip	81:23	2016	blood

**Table 2:** Top 20 up- and down-regulated DEGs

ID	Symbol	Combined.ES	***p***-value	FDR	Regulation
3429	IFI27	3.792026522	0	0	up
10,561	IFI44	1.976537999	0	0	up
10,410	IFITM3	1.935026901	0	0	up
10,964	IFI44L	1.868513043	0	0	up
3005	H1F0	1.862181187	0	0	up
51,246	SHISA5	1.859002522	0	0	up
53,831	GPR84	1.845295269	0	0	up
2209	FCGR1A	1.82735172	0	0	up
94,240	EPSTI1	1.806694757	0	0	up
4061	LY6E	1.780227418	0	0	up
2210	FCGR1B	1.764902124	0	0	up
7100	TLR5	1.749471365	0	0	up
1992	SERPINB1	1.723938152	0	0	up
2537	IFI6	1.718541654	0	0	up
9381	OTOF	1.691374197	0	0	up
2992	GYG1	1.688238715	0	0	up
56,729	RETN	1.687707715	0	0	up
383	ARG1	1.668080182	0	0	up
4001	LMNB1	1.651344954	0	0	up
6813	STXBP2	1.642842541	0	0	up
1953	MEGF6	−1.748916017	0	0	down
4209	MEF2D	−1.745899494	0	0	down
79,600	TCTN1	−1.745363269	0	0	down
115,352	FCRL3	− 1.673846149	0	0	down
29,997	GLTSCR2	− 1.636549497	0	0	down
4034	LRCH4	−1.622090653	0	0	down
2205	FCER1A	−1.581356037	0	0	down
9649	RALGPS1	−1.57368542	0	0	down
55,692	LUC7L	−1.56811747	0	0	down
3820	KLRB1	−1.550638661	0	0	down
2091	FBL	−1.534536985	0	0	down
23,368	PPP1R13B	−1.517967293	0	0	down
23,492	CBX7	−1.507789439	0	0	down
1938	EEF2	−1.50136634	0	0	down
2744	GLS	−1.495629886	0	0	down
5051	PAFAH2	−1.484535521	0	0	down
51,386	EIF3L	−1.467836674	0	0	down
6141	RPL18	−1.437451113	0	0	down
9252	RPS6KA5	−1.436515977	0	0	down
1408	CRY2	−1.434786164	0	0	down

*DEG* Differentially expressed gene, *ES* Effect size, *FDR* False discovery rate

### Functional annotation

**Fig. 2:**
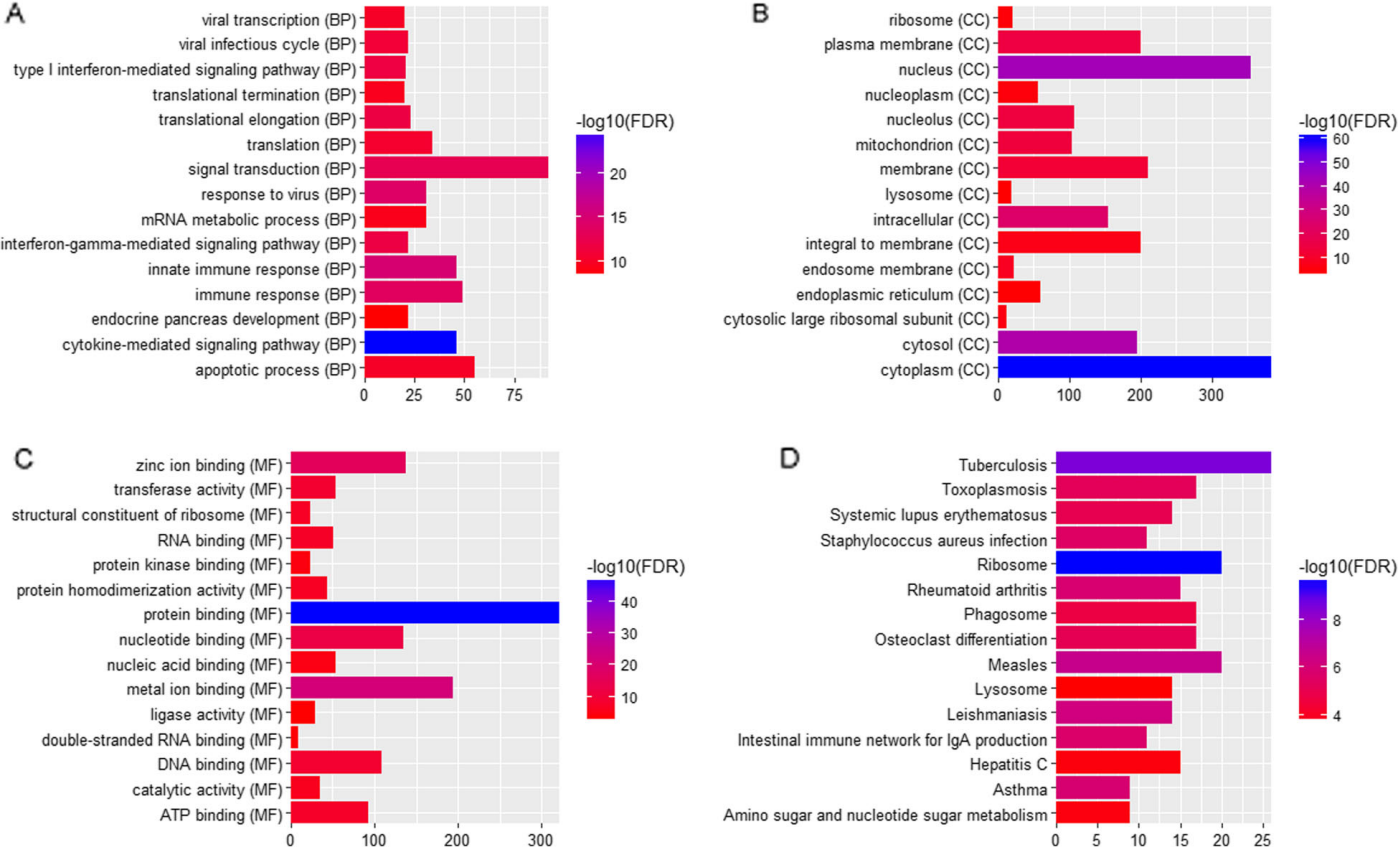
Go functional enrichment and KEGG analysis of DEGs in RSV (FDR < 0.05). **a** Biological process, **b** Cellular components, **c** Molecular functions. **d** KEGG analysis

**Fig. 3:**
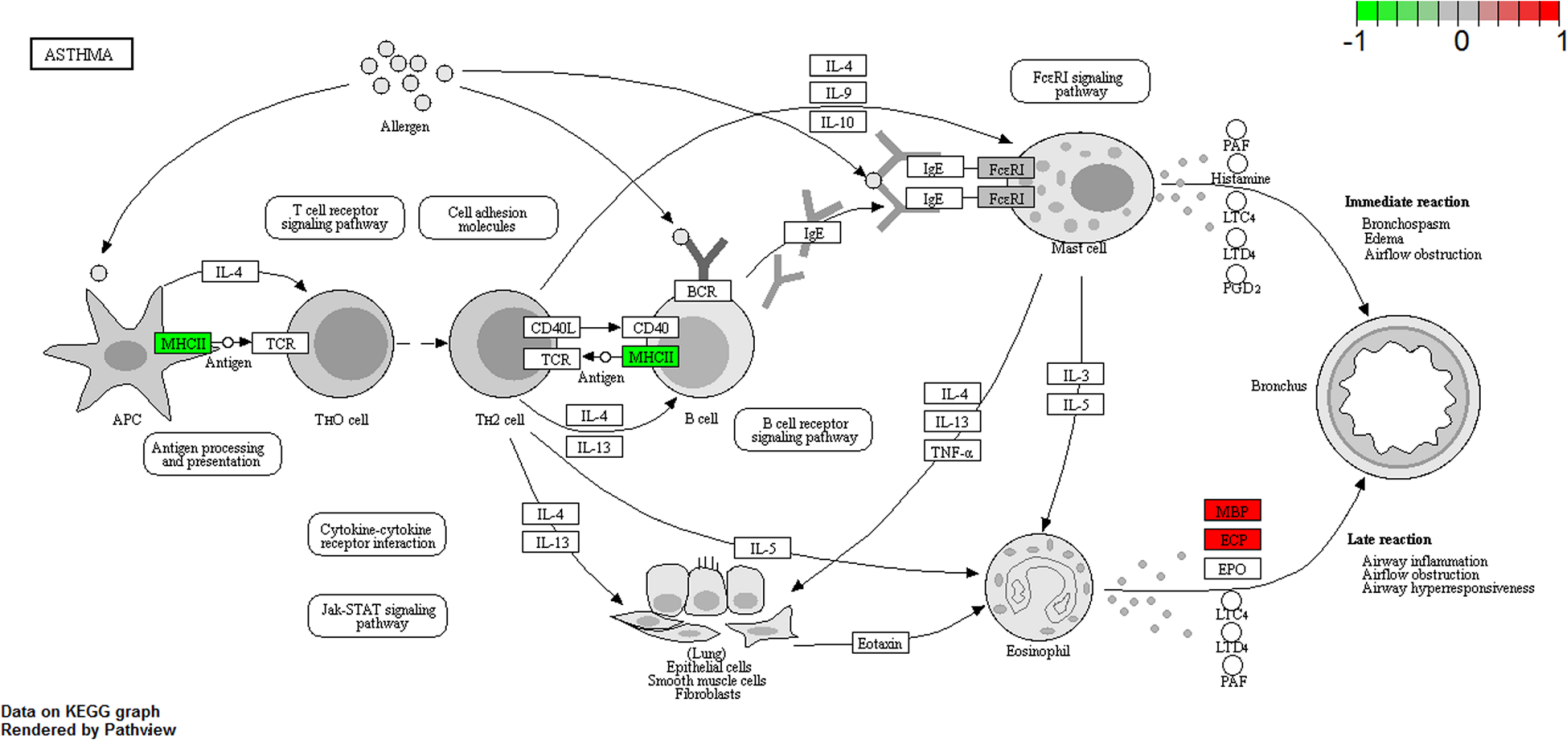
The details of ‘Asthma’ pathway

In Fig. 2a-c, GO enrichment revealed that the DEGs were significantly enriched in the biological processes of ‘cytokine-mediated signaling pathway’ (FDR = 1.17E-25), ‘innate immune response’ (FDR = 1.02E-15), ‘response to virus’ (FDR = 7.13E-15), the cellular components of ‘cytoplasm’ (FDR = 4.29E-61), ‘nucleus’ (FDR = 1.35E-44), ‘cytosol’ (FDR = 3.12E-41), ‘mitochondrion’ (FDR = 1.39E-14) and molecular functions of ‘protein binding’ (FDR = 3.90E-48), ‘metal ion binding’ (FDR = 4.42E-23) and ‘zinc ion binding’ (FDR = 2.34E-17). Furthermore, as shown in Fig. 2d, the results of KEGG pathway enrichment analysis revealed that DEGs were enriched in ‘Tuberculosis’ (FDR = 2.38E-09), ‘Measles’ (FDR = 2.29E-07), ‘Leishmaniasis’ (FDR = 5.62E-07) and ‘Asthma’ (FDR = 1.28E-06, Fig. 3).

### PPI network construction

**Fig. 4:**
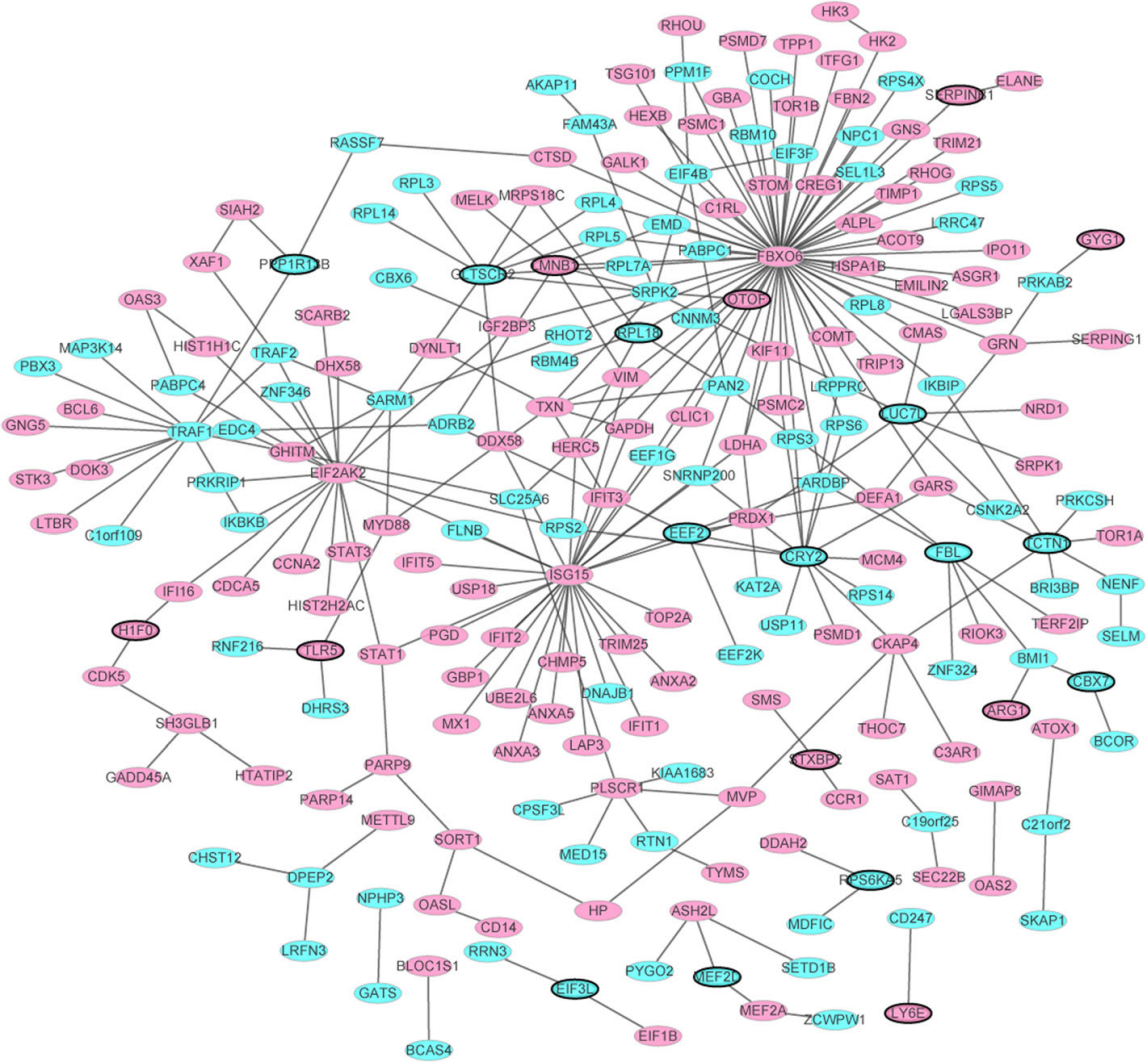
The PPI network of top 100 significantly DEGs in RSV. The green ellipses were represented the proteins encoded by down-regulated DEGs and the red ellipses were represented the proteins encoded by up-regulated DEGs. Among which, ellipses with black border were derived from the top 20 down-regulated DEGs in RSV

In Fig. 4, the PPI network consists of 229 nodes and 226 edges. Among them, the genes with higher degrees were FBXO6 (degree = 66), ISG15 (degree = 30), EIF2AK2 (degree = 19), CRY2 (degree = 13), TRAF1 (degree = 13), GLTSCR2 (degree = 9), TXN (degree = 7), TCTN1 (degree = 7), SRPK2 (degree = 7), EEF2 (degree = 6), LMNB1 (degree = 6), EIF4B (degree = 6), FBL (degree = 6), LUC7L (degree = 6), PLSCR1 (degree = 6). Among which, the three proteins of FBXO6, ISG15 and EIF2AK2 were hub proteins.

### TF regulatory network

**Fig. 5:**
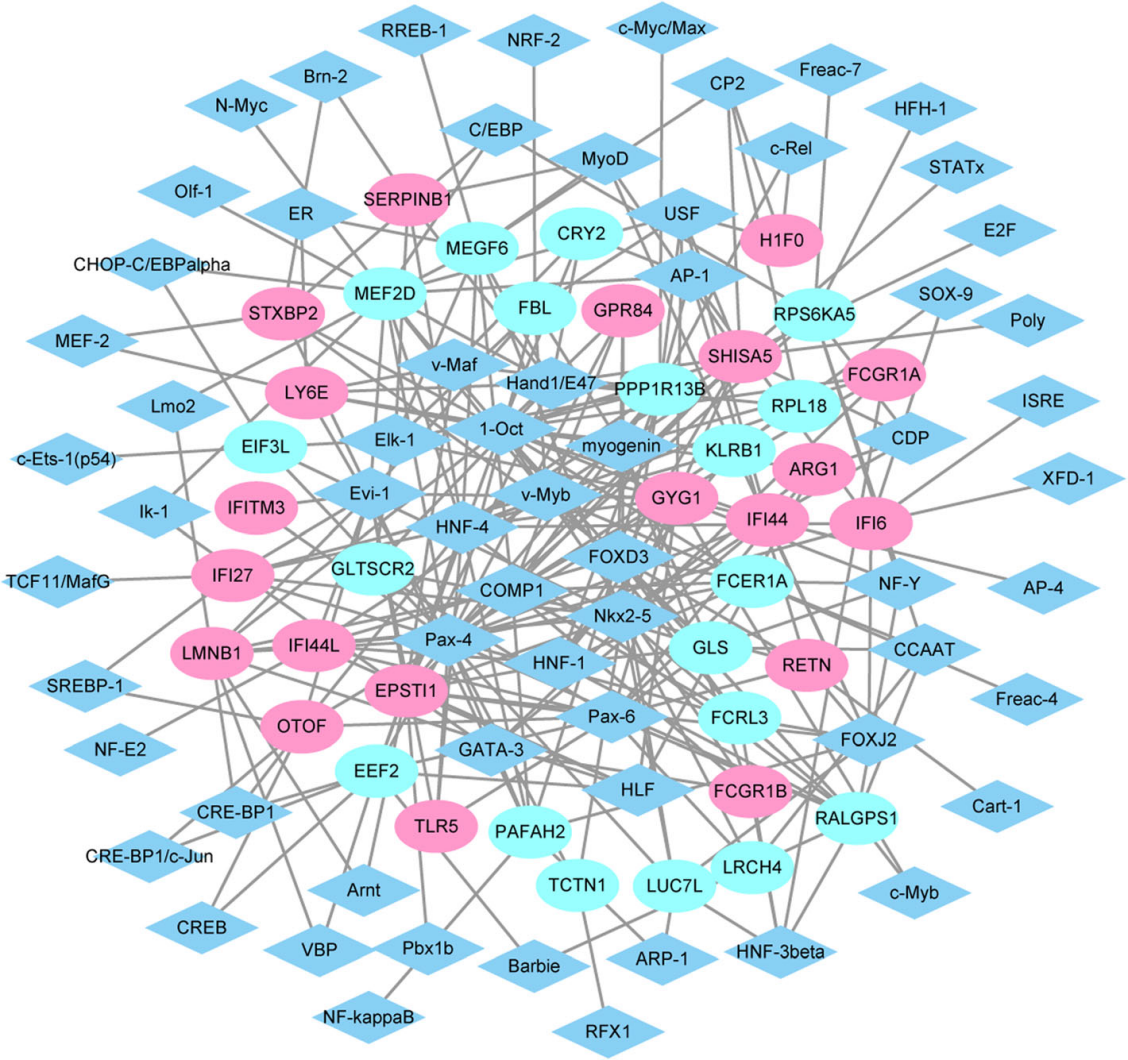
The RSV-specific transcription factors regulation network diagram. Blue diamond were represented transcription factors, ovals were represented top40 genes, red ovals were represented up-regulated genes, green ovals were represented down-regulated genes

TF regulatory network, which consists of 103 nodes and 287 edges, involving in 64 TFs and 39 DEGs, were obtained (Fig. 5). Among them, Pax-4, 1-Oct, Nkx2–5, HNF-4, COMP1, and Pax-6 were top 6 TFs with the most downstream genes (Table 3). The highest degree of 10 target genes were EPSTI1 (degree = 16), GYG1 (degree = 14), SHISA5 (degree = 14), PPP1R13B (degree = 14), MEF2D (degree = 13), RALGPS1 (degree = 12), MEGF6 (degree = 10), FCER1A (degree = 10), LMNB1 (degree = 10), EEF2 (degree = 10).

**Table 3:** The top 6 TFs with the most downstream regulatory genes and their target genes

TF	Number of regulated genes	Regulated genes
Pax-4	24	PAFAH2, FCRL3, FCER1A, IFI6, TLR5, MEF2D, IFI44, OTOF, SHISA5, GYG1, LMNB1, LY6E, RALGPS1, IFITM3, TCTN1, GPR84, EPSTI1, IFI27, RPS6KA5, PPP1R13B, LUC7L, FBL, EEF2, EIF3L
1-Oct	19	FCGR1A, FCGR1B, MEF2D, FCRL3, FCER1A, IFI44, GYG1, SHISA5, LMNB1, ARG1, LY6E, RALGPS1, CRY2, KLRB1, GPR84, EPSTI1, PPP1R13B, STXBP2, EEF2
Nkx2–5	18	MEGF6, IFI44, IFI44L, GYG1, SHISA5, LMNB1, ARG1, LRCH4, RALGPS1, TCTN1, GPR84, EPSTI1, IFI27, PPP1R13B, LUC7L, RETN, GLTSCR2, EIF3L
HNF-4	16	PAFAH2, MEF2D, IFI6, TLR5, MEGF6, OTOF, GYG1, SHISA5, SERPINB1, RALGPS1, KLRB1, EPSTI1, IFI27, PPP1R13B, RPL18, EEF2
COMP1	13	PAFAH2, FCGR1A, FCGR1B, FCRL3, MEGF6, FCER1A, GLS, SHISA5, LMNB1, GPR84, EPSTI1, PPP1R13B, RPL18
Pax-6	13	FCER1A, TLR5, IFI44L, GLS, OTOF, GYG1, ARG1, LRCH4, RALGPS1, EPSTI1, RETN, EEF2, GLTSCR2

### QRT-PCR confirmation

**Fig. 6:**
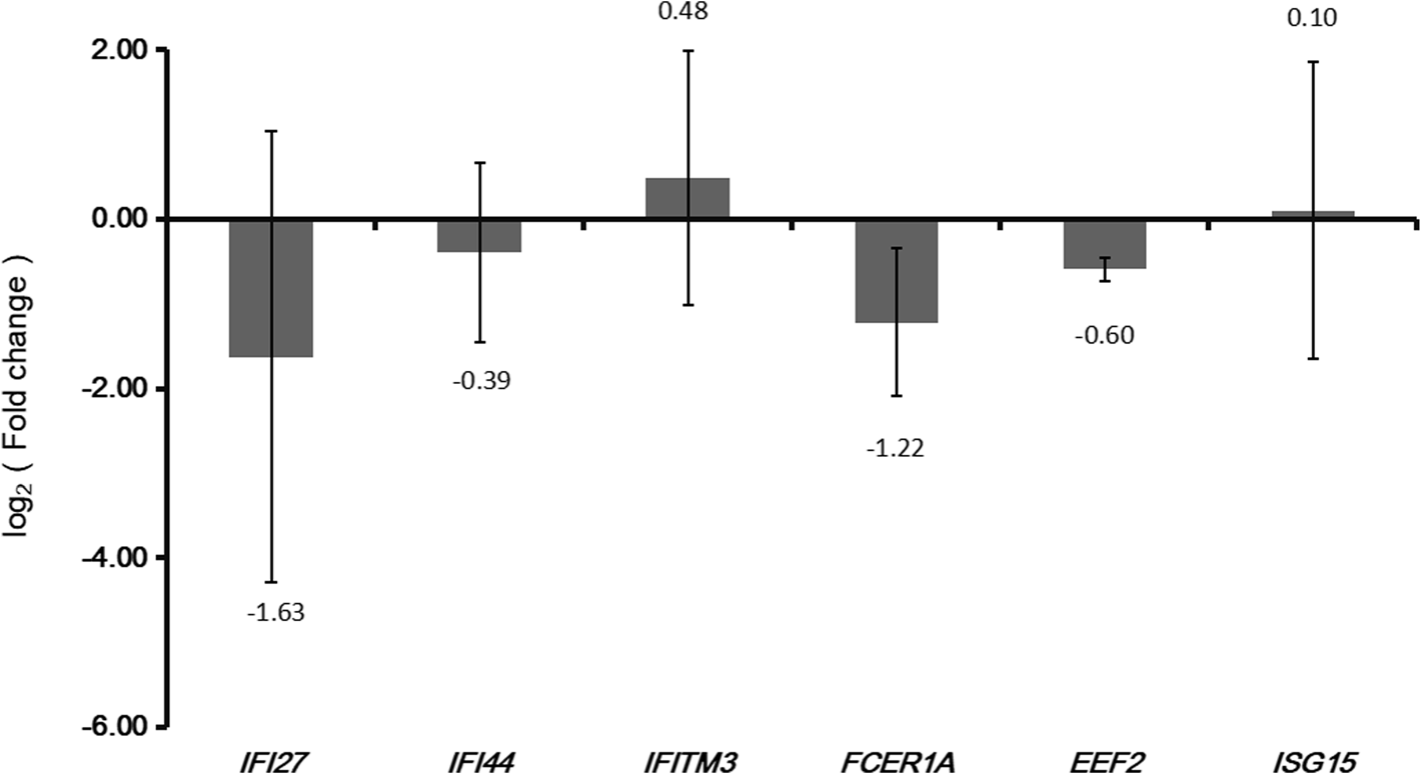
qRT-PCR results of DEGs in RSV. The X-axis represents the DEGs and the Y-axis represents the relative expression levels. * Indicates *p* < 0.05; ** Indicates *p* < 0.01; *** Indicates *p* < 0.001

Six genes, including IFI27, IFI44, IFITM3, FCER1A, EEF2 and ISG15, were selected to test by qRT-PCR. In Fig. 6, except for IFI27 and IFI44, the expression of IFITM3, FCER1A, EEF2 and ISG15 in qRT-PCR was consistent with our integrated analysis.

### Validation in the GEO dataset and ROC analysis

**Fig. 7:**
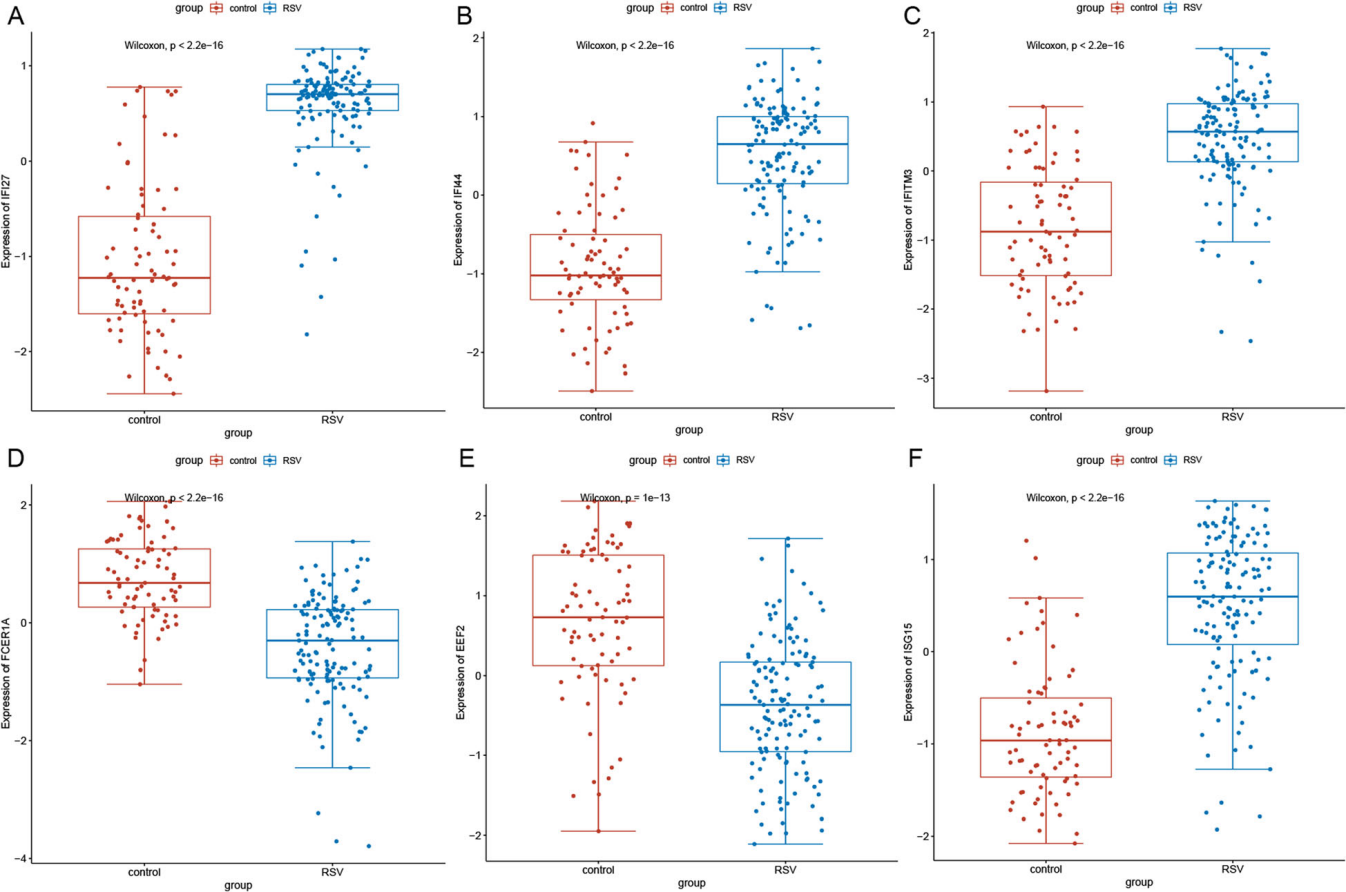
Validation of selected DEGs in GEO. The x-axes represent normal control and RSV groups. The y-axes represent gene expression levels. **a** IFI27, **b** IFI44, **c** IFITM3, **d** FCER1A, **e** EEF2, **f** ISG15

The expression patterns of six DEGs, including IFI27, IFI44, IFITM3, FCER1A, EEF2 and ISG15, were verified with GSE34205, GSE38900, GSE42026 and GSE105450. As shown in Fig. 7, IFI27, IFI44, IFITM3 and ISG15 were up-regulated, and FCER1A and EEF2 were down-regulated in RSV, which were consistent with our integrated analysis.

**Fig. 8:**
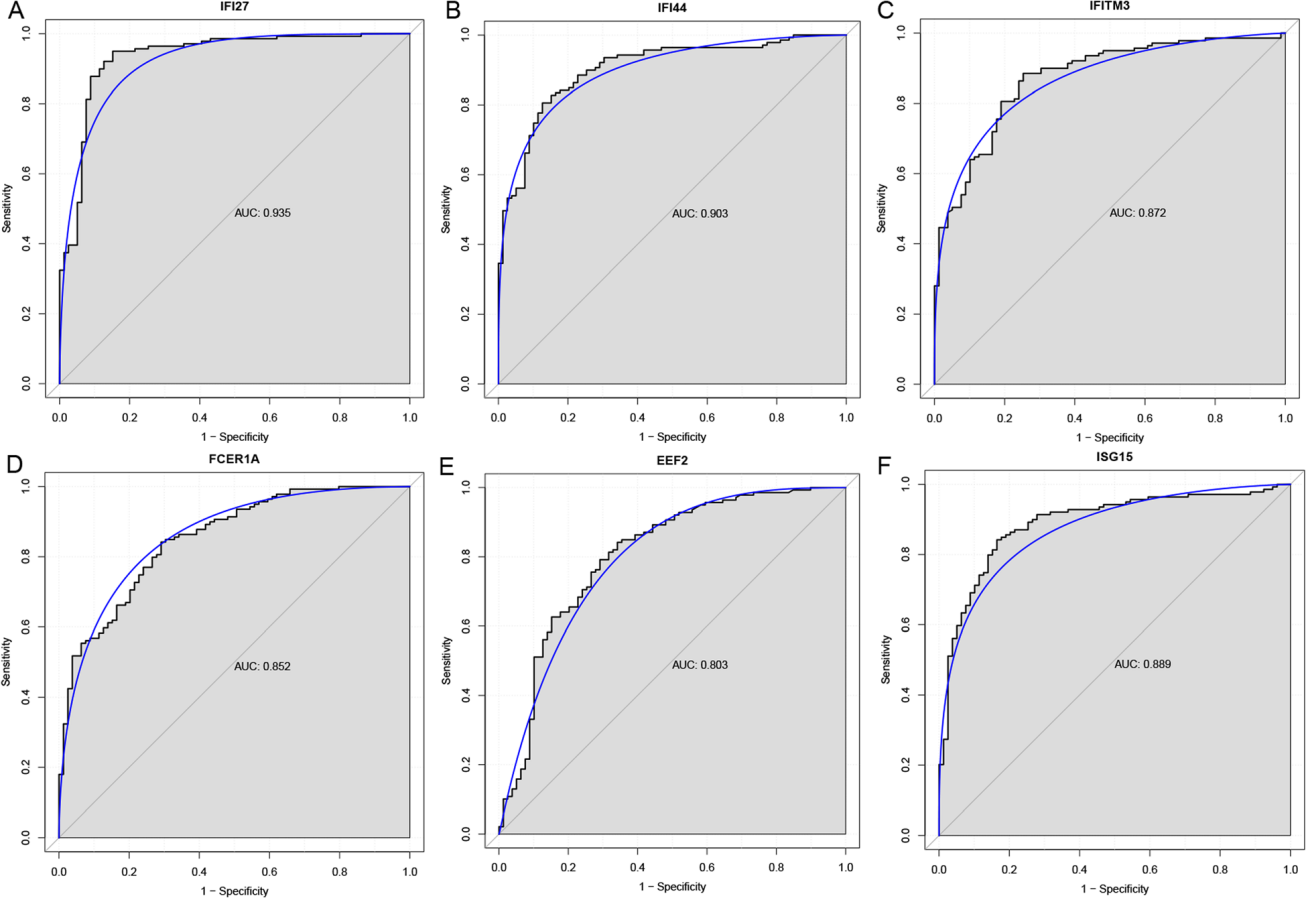
The ROC curves of DEGs in RSV. The ROC curves were used to show the diagnostic ability of these selected DEGs with sensitivity (the proportion of true positive) and 1-specificity (the proportion of false positive). The x-axis shows 1-specificity and y-axis shows sensitivity. **a** IFI27, **b** IFI44, **c** IFITM3, **d** FCER1A, **e** EEF2, **f** ISG15

We performed ROC curve analyses and calculated the AUC to assess the diagnostic value of these six DEGs. The AUC of these six DEGs, including IFI27 (0.935), IFI44 (0.903), IFITM3 (0.872), FCER1A (0.852), EEF2 (0.803) and ISG15 (0.889), was more than 0.80, which indicated that these six DEGs were with diagnostic value (Fig. 8).

## Discussion

RSV is the most common viral pathogen causing acute lower respiratory tract infections in infants, children and older people [10]. In this study, we performed an integrated analysis using data obtained from the GEO database. KEGG, GO and other biological information databases, and R analysis tools were used to analyze the DEGs. We obtained 1059 DEGs in RSV (493 genes were up-regulated, 566 genes were down-regulated). We also identified important signaling pathways that affect the pathogenesis of RSV such as ‘response to virus’ and ‘Asthma’. In addition, based on the promoter sequence of DEGs obtained from UCSC, a TF regulatory network was constructed using the match tool of the TRANFAC website to obtain the corresponding TFs.

IFI27 is a hydrophobic mitochondrial protein composed of 122 amino acid [11]. IFI27 belong to a group of small interferon stimulated genes (ISGs) [12, 13]. Rosebeck and Leaman et al. reported that IFI27 maintains a low background expression in various mammalian cells and participates in a variety of biological processes, including apoptosis and congenital immunity [14, 15]. IFI27 expression was elevated in the psoriatic lesions and uterine fibroids, ovarian cancer, and other diseases [16, 17]. It has also been shown to have a direct antiviral effect against certain viruses [18]. Hans-Olav Fjaerli et al. reported the gene IFI27 is up-regulated in whole blood of infants hospitalised with RSV [19]. According to our study, up-regulated IFI27 was among the top 20 differentially expressed mRNAs and was enriched in the GO item mitochondrion (FDR = 1.39E-14).

IFI44 is a member of the type I interferon-inducible gene family. Microtubule-associated protein 44 (IFI44) has been reported to be antiproliferative [20]. IFI44, also termed interferon-inducible protein 44 or p44 as it aggregates to form microtubular structures, is part of the type I IFN-inducible gene family. Its promoter region contains an IFN-α stimulation responsive elements, which can mediate type I IFN-inducible gene pathway [21]. Jacqueline U. McDonald et al. identified IFI44 gene serve as potential targets for future investigation in RSV disease [22]. In our study, IFI44 was up-regulated and among the top 20 differentially expressed mRNAs, which support the previous researches. Furthermore, IFI44 was enriched the GO term response to virus (FDR = 7.13E-15).

IFITM3 is a member of the interferon-inducible transmembrane protein family, which play a role in regulating antiviral signaling, inflammation, and somatogenesis [23]. In the IFITM3 knockout mouse model, IFITM3 has been reported to inhibit RSV cell infection and control the pathogenesis of the disease [24]. In our integrated analysis, IFITM3 was up-regulated and among the top 20 differentially expressed mRNAs.

The Fc fragment of IgE, a high affinity I, is a receptor for alpha polypeptides, also known as FCER1A, a protein encoded by the FCER1A gene in humans [25]. High-affinity IgE receptors play an important role in allergic diseases, coupled allergens, and mast cells, triggering inflammation and immediate allergic reactions, which are characteristic of diseases such as hay fever and asthma. Infants with severe RSV infections will subsequent develop asthma later during childhood [26]. In the KEGG analysis, the item of ‘Asthma’ (FDR = 1.28E-06) was significantly enriched and the down-regulated FCER1A was enriched in this pathway. In addition, FCER1A was among the top 20 differentially expressed mRNAs. Moreover, in the transcription factors regulation network, FCER1A (degree = 10) was among the top 10 targeted genes with high degree.

IFN-stimulated genes (ISGs) produce an antiviral state that plays an important role in determining host innate and adaptive immune responses [27]. One of the most highly induced genes in the IFN response is ISG15, which encodes a 17 kDa small UBL protein that forms a covalent conjugate with cellular proteins that mediate a large number of antiviral responses [28, 29]. Rubén González-San et al. found that ISG15 is up-regulated in respiratory pseudostratified epithelial cells and infant nasopharyngeal lavage fluids infected with RSV [30]. In our results, ISG15 was up-regulated and was the hub protein in the PPI network.

## Conclusion

In conclusion, five DEGs (IFI27, IFI44, IFITM3, FCER1A, and ISG15) were identified to be involved in RSV. From the three GEO datasets analyzed, we identified 1059 DEGs (493 up-regulated and 566 down-regulated genes) between RSV and normal controls. Our findings may contribute to the elucidation of new potential biomarkers, reveal the underlying pathogenesis and identify novel therapeutic targets for the treatment of RSV. Our study also had limitations. The samples used for study was blood samples for a mucosa-limited infection disease, and no functional experiments was performed to validate the results. To confirm the exact function of the biomarkers found in this study, more samples will be collected and more in deep research on functional experiments will be included in our future work.

## Supplementary Information

**Additional file 1: Figure S1.** PCA of three datasets used in this study.

**Additional file 2: Table S1.** Clinical features of patients with RSV and controls.

## Data Availability

The datasets used and/or analysed during the current study available from the corresponding author on reasonable request.

## Abbreviations

RSV: Respiratory syncytial virus
TF: Transcription factor
DEG: Differentially expressed gene
GEO: Gene Expression Omnibus
FDR: False discovery rate
PPI: Protein-protein interaction
UCSC: University of California Santa Cruz
ROC: Receiver operating characteristic
AUC: Area under the curve
PCA: Principal component analysis
ISG: Interferon stimulated gene
