# Supplementary figures and images for: Identification of pathogenic genes and transcription factors in respiratory syncytial virus
Source: BMC Pediatr. 2021 Jan 8;21:27. doi: 10.1186/s12887-020-02480-4 (PMC7796524; doi:10.1186/s12887-020-02480-4)

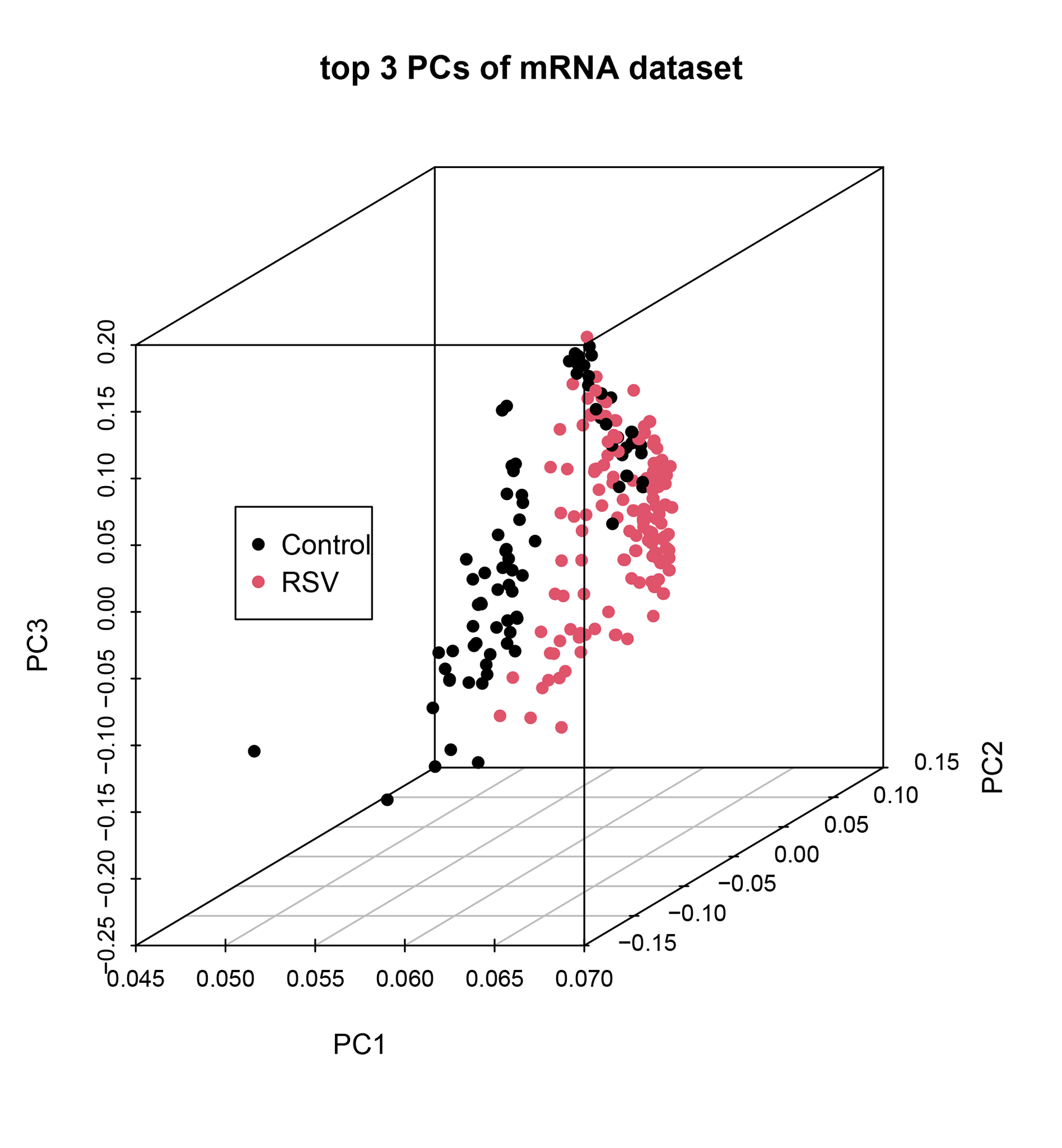

Supplement: Supplementary file 1 — Additional file 1: Figure S1. PCA of three datasets used in this study. [file 12887_2020_2480_MOESM1_ESM.tif]
