# Supplementary material for: Identification of pathogenic genes and transcription factors in respiratory syncytial virus
Source: BMC Pediatr. 2021 Jan 8;21:27. doi: 10.1186/s12887-020-02480-4 (PMC7796524; doi:10.1186/s12887-020-02480-4)
Supplement: Supplementary file 2 — Additional file 2: Table S1. Clinical features of patients with RSV and controls. [file 12887_2020_2480_MOESM2_ESM.docx]

**Table S1 Clinical features of patients with RSV and controls**

|  | Age (years) | Weight (kg) | RSV  (throat swab) | WBC (10^9^/L) | Neutrophils (%) | Lymphocyte (%) | Monocyte (%) | Eosinophilic granulocyte (%) | Hemoglobin (g/L) | Platelets (g/L) |
| --- | --- | --- | --- | --- | --- | --- | --- | --- | --- | --- |
| Case 1 | 2-5.2 | 14.2 | positive | 6 | 48.9 | 40.4 | 6.4 | 4.3 | 112 | 467 |
| Case 2 |  | 31 | positive | 8.71 | 67.9 | 27.4 | 4.1 | 0.6 | 104 | 509 |
| Case 3 |  | 21 | positive | 7.56 | 26.7 | 70.3 | 2.3 | 0.7 | 121 | 518 |
| Control 1 | 7-11.8 | 30.5 | - | - | - | - | - | - | - | - |
| Control 2 |  | 35 | - | - | - | - | - | - | - | - |
| Control 3 |  | 36 | - | - | - | - | - | - | - | - |

RSV, respiratory syncytial virus; WBC, White blood cells.
